# Survivability of Microbes in Natural Environments and Their Ecological Impacts

**DOI:** 10.1264/jsme2.ME3002rh

**Published:** 2015-06

**Authors:** Shin Haruta, Nanako Kanno

**Affiliations:** 1 Graduate School of Science and Engineering, Tokyo Metropolitan UniversityMinami-Osawa 1–1, Hachioji, Tokyo 192–0397Japan

Microbiology often focuses on the growth properties or physiological capabilities of microbes. However, most microbes in the natural environment are not actively growing. As has been proposed for plants, microbial life strategies should consider stress tolerance as well as competitive relationships and physiological and biological disturbance (19). Reports studying microbial communities in oligotrophic or nutrient-limited environments have increased recently, *e.g.* (10), (21), and (41). How do microbes live? Do they mostly simply do nothing? Recent reports on microbial survivability, introduced in this Research Highlight, are providing informative answers for these questions, and discovering new aspects of microbial ecosystems.

## Response to continuous, simultaneous or successive stresses

Microbes must overcome various stresses that suppress their ability to grow or their basic survival. Numerous strategies exist in bacteria to cope with stressful conditions including the formation of cysts and spores, changes in cellular membranes, expression of repair enzymes for damage, synthesis of molecules for relieving stresses, and so forth (38).

How do environmental conditions affect the stress susceptibility of microbes? Continuous stress is a selective pressure and may induce genetic modification. For example, Itoh *et al.* found a strain of *Nitrosomonas* from a thermal environment that shared 100% identity in its 16S rRNA gene sequence with the mesophilic *Nitrosomonas nitrosa* Nm90, but its growth temperature was expanded to 48°C (13). On the other hand, bacteria under no selective pressures sometimes show tolerance against chemicals, they are apparently naïve to. *Legionella* that are highly tolerant against the biocide monochloramine have been isolated from water systems where this chemical had not been previously applied (14).

Some environmental bacteria intrinsically possess multiple survival strategies against occasional exposures to stresses, since bacteria often face combinations of stresses simultaneously. As an example, one extreme environment microbes survive in is the built environment. Yano *et al.* showed multi-stress tolerance in *Methylobacterium* isolated from bathrooms, which are characterized by rapid water flow, frequent cycles of wet and dry, limited available nutrients, occasional exposure to cleaning agents and so on (43). Multi-stress tolerance may be achieved by global regulation of multiple genes related to stress tolerances, *e.g.*, sigma factors and chaperons (20, 38). A recent study reported that a chaperonin, GroEL2, in *Rhodococcus* sp. mediated tolerance against an organic solvent through changes in growth rate, cell surface structure, *etc*. (39).

These adaptive mechanisms can also be effective for successive stresses. Effects of starvation on susceptibility to antibiotics have been well-documented (23, 30). Recently, cold- or heat-stress induced changes in stress tolerance and virulence were reported for pathogenic bacteria (17, 36). These reports tried to simulate typical conditions used for pathogen control, *i.e.*, heat treatment and food storage at low temperatures. Furthermore, co-existing bacteria can also impose stresses. A notable example was reported by Müller *et al. (*28). They found that *Bacillus* responded to a stress caused by predation from *Myxococcus* by altering its cell morphology and producing a toxin against the predator. Natural environments are permanently and frequently changing worlds for microbes. Studies on microbial responses to simultaneous and successive stresses will be informative with respect to how bacteria persist in nature.

## Non-dividing but metabolically active state

Microbes in a senescent state do not seem to cease all metabolic activity, but rather they keep partially metabolically active in order to maintain viability and protect against stress conditions. Such reduced metabolism however, still necessitates appropriate amounts of energy. Various approaches have been conducted to estimate the minimal maintenance energy in stable and nutrient-limited environments (9). Koenig *et al.* indicated an energy requirement for response to stresses, because bacterial cells under lower energy-producing conditions were more sensitive to toxic solvents (18). In contrast, photosynthetic energy production in the light increased the viability of photosynthetic bacteria under nutrient-limited conditions (7, 15, 35). Through the action of proteorhodopsin, a light-driven proton pump, light energy is also converted to chemical energy required for starvation survival (1, 6).

Cells in a non-dividing but metabolically active state are likely dormant and sometimes require a signal to awake. For example, pyruvate worked in the resuscitation process of *Salmonella* (26). In the phylum *Actinobacteria*, the resuscitation promoting factor (Rpf) protein demonstrated both a resuscitating ability and a growth promoting activity (27, 32, 33). However, it is challenging to clarify environmental cues related to microbial activity, and how microbes in non-dividing but metabolically active states sense these cues to decide whether or not initiate active growth. Stochastic transcriptional control is another possible mechanism underlying the occasional awakening of a part of the population independent of environmental cues (3).

## Alternative survival strategies

Chemotactic behaviors, *i.e.* moving toward attractants and away from repellents, are apparently an effective set of survival strategies in heterogeneous environments. For some bacteria, migration towards the rhizosphere of plants is the most promising way to obtain organic nutrients in soils; tomato root colonization of a plant growth-promoting rhizobacterium, *Pseudomonas fluorescens* (2, 31). However, future research should clarify whether or not if the speed and distances traveled via bacterial cellular motility are practical such that cells can advantageously reach roots in soil environments.

Programmed cell death in bacteria has also been proposed as a possible strategy for preserving some of the population from stresses through utilization of the nutrients derived from the dead cells (34). The effectiveness of such programmed cell death in bacteria as a survival strategy is however a highly controversial issue in microbial ecology.

## Ecological impacts of microbial survivability

Survivability of pathogenic bacteria is a crucial issue in public health, particularly with respect to hospitals. Development of detection methods of such bacteria has been intensively studied as has been the physiological characterization of pathogens in environmental conditions. Inoue *et al.* applied an ethidium monoazide and quantitative PCR (EMA-qPCR) method to successfully find a wide diversity of viable *Legionella* from cooling tower waters (11). An outstanding mini-review by Martínez-Vaz *et al.* showed the life strategy of pathogenic bacteria during their long path from native soil into the rhizosphere and phyllosphere of agricultural plants and then into the food supply (24). Furthermore, recent studies are clarifying the survivability of viruses and protozoa (4, 5, 37). Such studies help to fully describe the microbial world. Recent research has further expanded our knowledge to include the survivability of microbes beyond our planet Earth (40, 42).

It is generally suggested that biodiversity-ecosystem functioning shows positive relationships. Hobbie and Hobbie reported that environmental microbes with low activity quickly responded to available nutrients (8). Non-dividing microbial populations are effectively part of a microbial seed bank (22). A recent review article proposed that limited diversity of ammonia oxidizers in soil may essentially determine stability of N-cycling functions within the ecosystem (12). In biotechnological fields, stress response and survivability of microbes are recognized as key factors relevant to the ability to maintain the functional stability of bioreactors (16, 29). It is noteworthy that a recent metagenomic study on composting processes revealed the abundance of genes related to stress responses (25). Stress response may promote stable ecosystem functioning by allowing for individual component taxa to persist under suboptimal conditions.

Methodological limitations mean we have not completely detected microbes under stressed and non-dividing conditions within microbial communities. Further studies are underway to clarify the physiological characteristics of non-dividing cells and their responses to stresses or resuscitation. These collective efforts will reveal the larger and wider ecological impacts of microbial survivability.
